# The role of dysregulated ghrelin/LEAP-2 balance in eating disorder: a translational study in anorexia nervosa.

**DOI:** 10.1192/j.eurpsy.2023.54

**Published:** 2023-07-19

**Authors:** C. Tezenas du Montcel

**Affiliations:** ^1^Université Paris Cité UMR-S 1266, Institut de Psychiatrie et Neurosciences de Paris, 75014 Paris; ^2^Clinique des Maladies Mentales et de l’Encéphale, GHU Paris Psychiatrie et Neurosciences Hôpital Sainte Anne, F-75014 Paris, France

## Abstract

**Abstract:**

The ghrelin system is a key regulator of appetite and food intake across species. LEAP-2, a recently discovered ghrelin antagonist, appears to be up-regulated in obesity and opposes to the orexigenic drive of ghrelin. The evolution of LEAP-2 levels could be an interesting insight to reflect the regulation of appetite in eating disorders such as anorexia nervosa (AN). We provide the first study exploring the ghrelin and LEAP-2 regulation in long-term food restriction followed by refeeding in both mice and patients suffering from AN.

Using a translational strategy, we compared the regulation of ghrelin and LEAP-2 concentrations in blood during food restriction and after refeeding in female mice exposed to a 14 days protocol combining quantitative food restriction and running wheel activity followed by progressive refeeding. We compared these results to clinical data fom in an ongoing longitudinal study of patients with AN evaluated before and after refeeding as well as 6 months after hospital discharge.

Long-term food restriction in mice was associated with increased ghrelin and decreased LEAP-2 concentrations compared to *ad libitum* fed controls. Refeeding led to an increase in LEAP-2 concentrations. Patients with AN displayed increased ghrelin levels but also higher LEAP-2 concentrations on admission than after refeeding. LEAP-2 decreased with refeeding. On 17 patients re-evaluated 6 months after discharge, patients with unstable weight gain exhibited a greater decrease of LEAP-2 concentrations during refeeding compared to patient with stable weight gain. Decreasing LEAP-2 concentrations was able to predict a negative outcome (i.e. unstable weight gain) in 80% of the cases.

We provide evidence that the ghrelin/LEAP-2 system is not regulated according to the nutritional status in AN as it is in the case of a physiological adaptation to food restriction. Our clinical data suggest that the evolution of LEAP-2 concentrations during refeeding is opposed to data from preclinical model and could give new insights on the outcome of weight gain in AN.

**Disclosure of Interest:**

None Declared

